# Supplementary material for: Triple-helix potential of the mouse genome
Source: Proc Natl Acad Sci U S A. 2022 May 3;119(19):e2203967119. doi: 10.1073/pnas.2203967119 (PMC9171763; doi:10.1073/pnas.2203967119)
Supplement: Supplementary File [file pnas.2203967119.sapp.pdf]

PNAS

Supplementary Information for:

Triple-helix potential of the mouse genome

Kaku Maekawa, Shintaro Yamada, Rahul Sharma, Jayanta Chaudhuri, and Scott Keeney

Scott Keeney
Email: s-keeney@ski.mskcc.org

This PDF file includes:

Figures S1 to S6
Tables S1 to S2

Other supplementary materials for this manuscript include the following:

Dataset S1

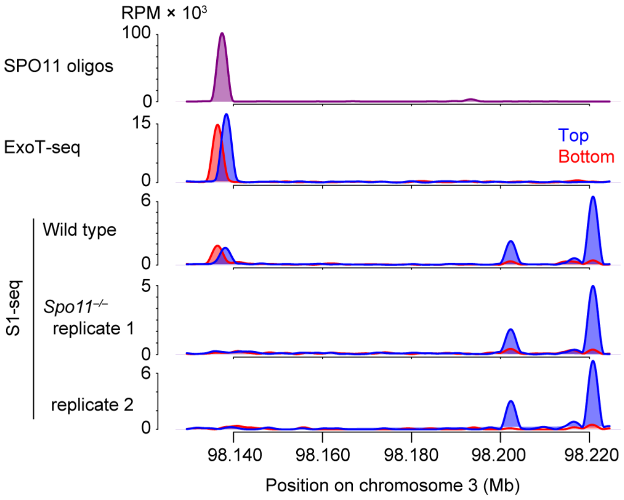

Fig. S1. Additional examples of SPO11-independent S1-seq clusters.

Strand-specific S1-seq at another representative DSB hotspot (left side, coincident with a peak in the SPO11-oligo sequencing) with two reproducible SPO11-independent read clusters nearby (right side).

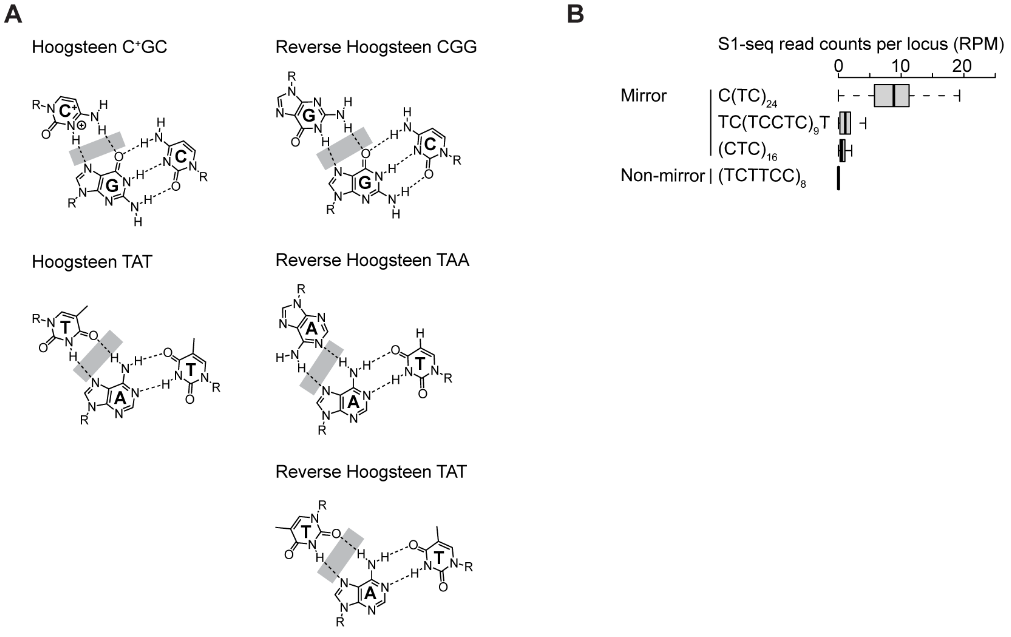

Fig. S2. Hoogsteen base pairs and S1-seq counts at pyrimidine repeats.

(**A**) Hoogsteen base pairs ($C^{+}GC$ and TAT) and reverse Hoogsteen base pairs (CGG, TAA, and TAT). H-y isomers are composed of Hoogsteen base pairs and H-r isomers are composed of reverse Hoogsteen base pairs. The dotted lines indicate hydrogen bonds; the gray rectangles highlight the bonds in Hoogsteen pairs.

(**B**) Comparison of *Spo11*$^{-/-}$ S1-seq counts at individual polypyrimidine mirror and non-mirror repeats. Boxes indicate median and interquartile range; whiskers indicate the most extreme data points which are 1.5 times the interquartile range from the box; outliers are not shown.

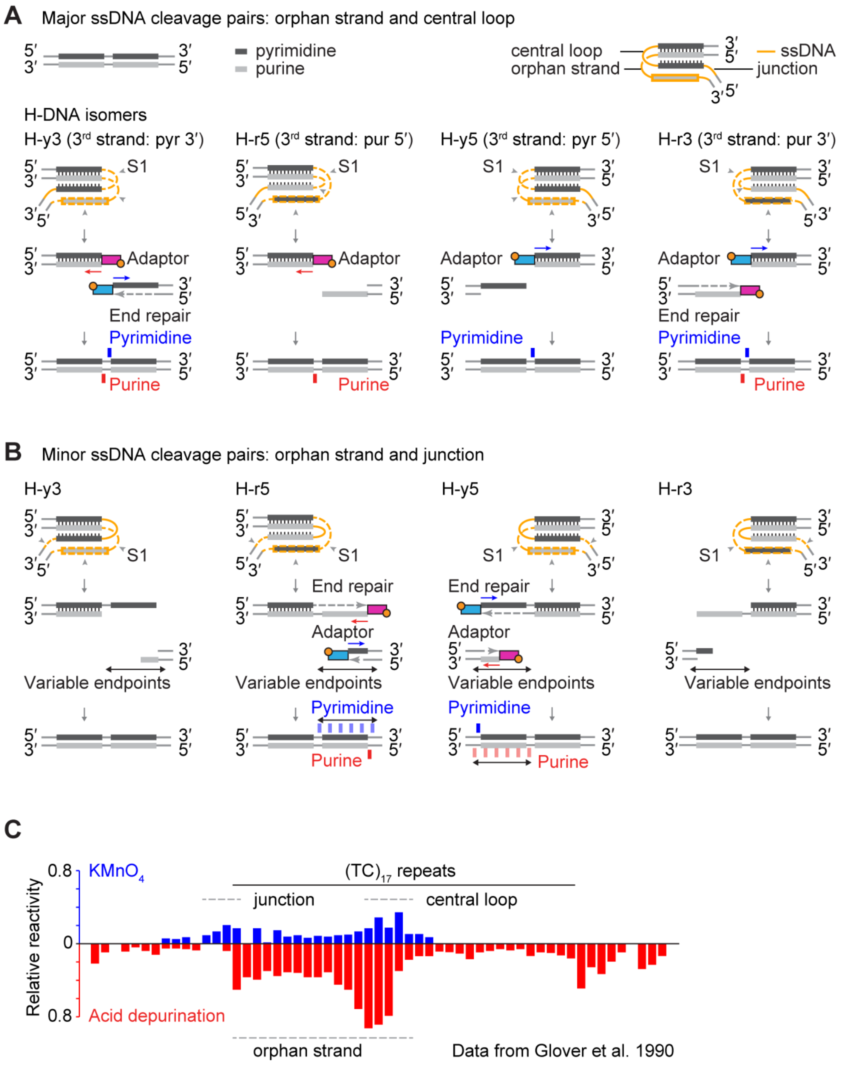

Fig. S3. Incomplete digestion of ssDNA in H-DNA can account for observed S1-seq patterns at C(TC)$_{20}$ sequences.

As in Fig. 3, in all panels, gray arrowheads indicate which ssDNA segments are digested with nuclease S1, and the adaptors are color coded to indicate whether the resulting sequencing read will map to the pyrimidine strand (blue) or purine strand (red). At the bottom of each schematic, the expected mapping position and strand for the S1-seq read(s) are shown.

(**A**) Predicted S1-seq patterns if the central loop and orphan strand (but not junction strand) of each H-DNA isomer is digested with nuclease S1. In this scenario, all four isomers are predicted to yield a central read (pyrimidine strand for H-y5 and H-r3; purine strand for H-y3 and H-r5). In addition, digestion of H-r3 or H-y3 would leave a 5′ overhang that, after fill-in with T4 DNA polymerase and ligation to a sequencing adaptor, is predicted to yield a second central read mapping to the opposite strand. In contrast, digestion of H-y5 or H-r5 would yield a 3′ overhang, which if

inefficiently polished would not yield any S1-seq read. If this scenario reflects the major S1 digestion pattern, it would explain why central reads are more abundant than junction reads.

(**B**) Predicted S1-seq patterns if nuclease S1 cleaves the junction strand, digests the orphan strand to leave variable end points, and fails entirely to digest the central loop. In this scenario, H-y3 and H-r3 would yield two DNA ends with 3′ overhangs. If these were inefficiently polished, no S1-seq read would result, as shown; if polished, they would yield reads indistinguishable from complete cleavage as shown in Fig. 3*C*. In contrast, H-y5 and H-r5 would yield 5′ overhangs that, after fill-in with T4 DNA polymerase, would yield a junction read plus an opposite-strand read inside the mirror repeat. For H-y5, this would yield a pyrimidine-strand junction read and purine-strand reads within the left half of the mirror repeat, consistent with the striated S1-seq signal observed (Fig. 1 *G* and *H*).

(**C**) Chemical sensitivity of a plasmid-borne H-y5-forming sequence, $(TC)_{17}$ (data from ref. 51).

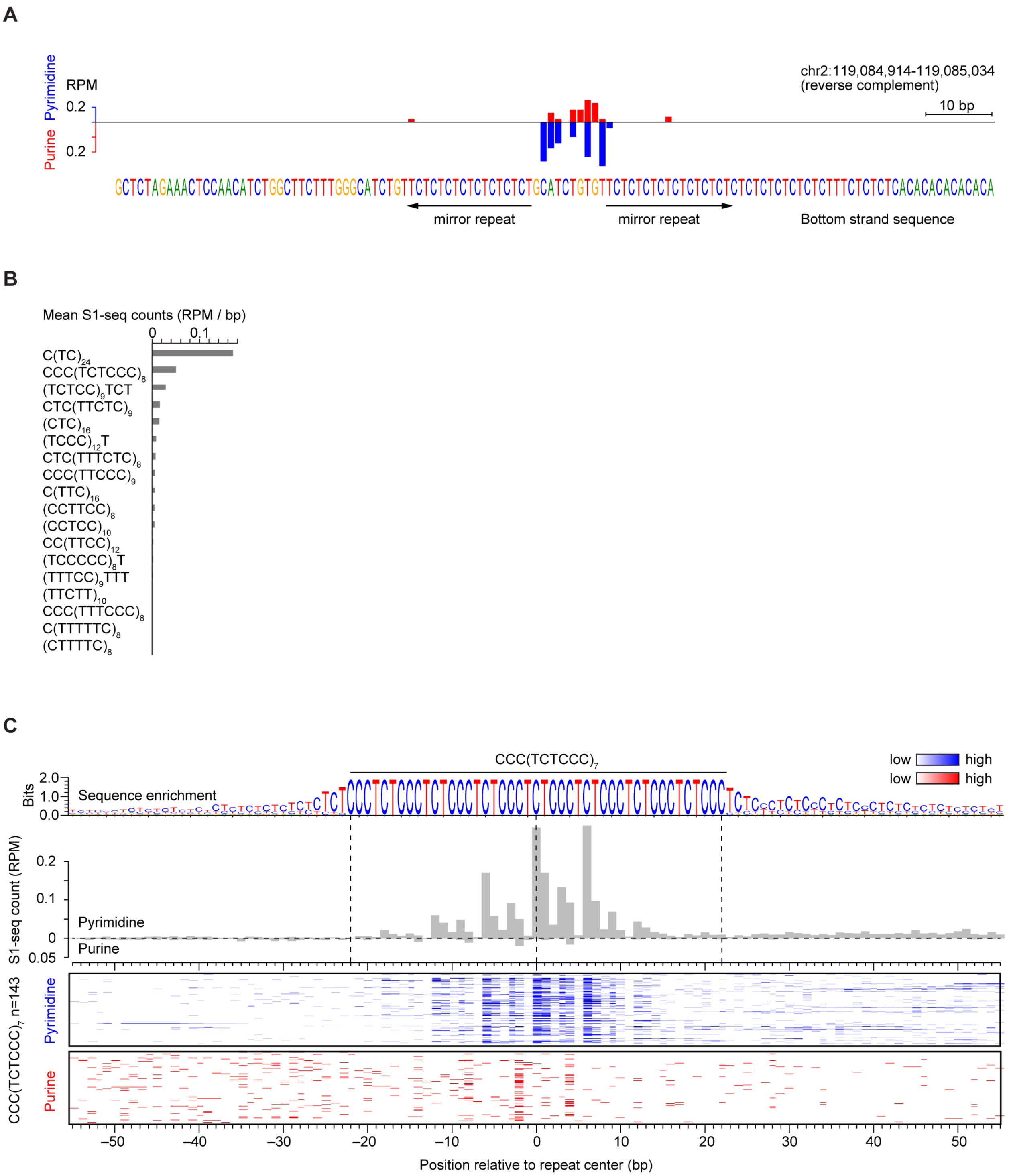

Fig. S4. Additional examples of S1-seq patterns around mirror repeats

(**A**) Unusual S1-seq read distribution at a pyrimidine mirror repeat with a 10-bp interruption between the repeats. In this example, roughly equal numbers of central reads were observed on both strands.

(**B**) Mean *Spo11*$^{-/-}$ S1-seq read densities at various polypyrimidine mirror repeats.

(**C**) S1-seq signal at CCC(TCTCCC)$_7$. Note that the fine-scale spatial pattern is distinct from C(TC)$_{20}$ (Fig. 1*H*), but is highly stereotyped across individual copies of this repeat.

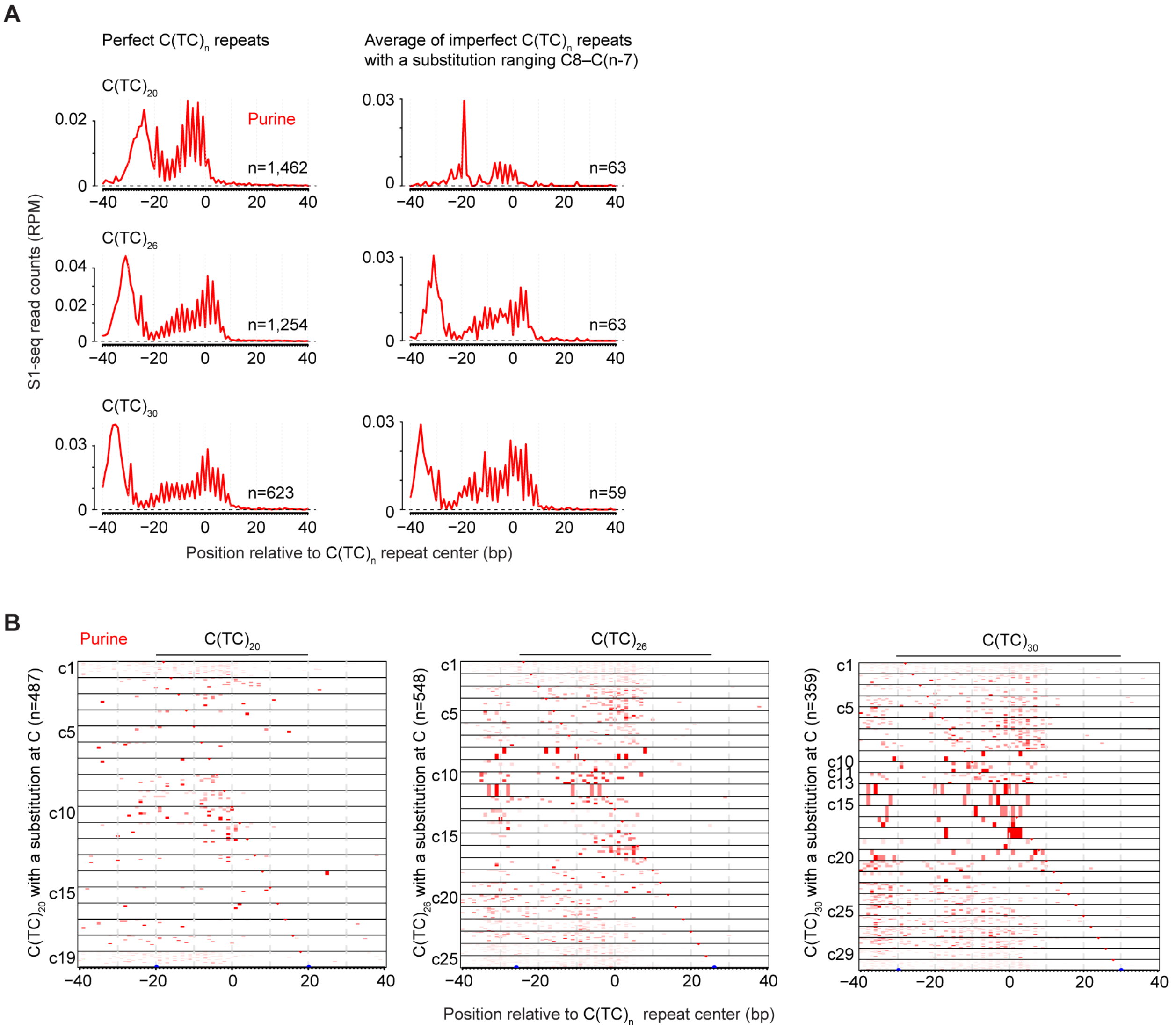

Fig. S5. Purine-strand S1-seq maps around imperfect C(TC)$_n$ repeats of various lengths.

(**A**) Averaged purine-strand S1-seq signal for C(TC)$_n$ repeats of the indicated lengths. The left graphs show signal for perfect repeats (same repeats as shown in Fig. 4*D*); the right graphs show signal for imperfect repeats (same repeats as shown in Fig. 4*H*).

(**B**) Heat maps of purine-strand S1-seq maps for imperfect C(TC)$_{20}$, C(TC)$_{26}$, and C(TC)$_{30}$ repeats. These are the same repeats shown in Fig. 4 *E*–*G*.

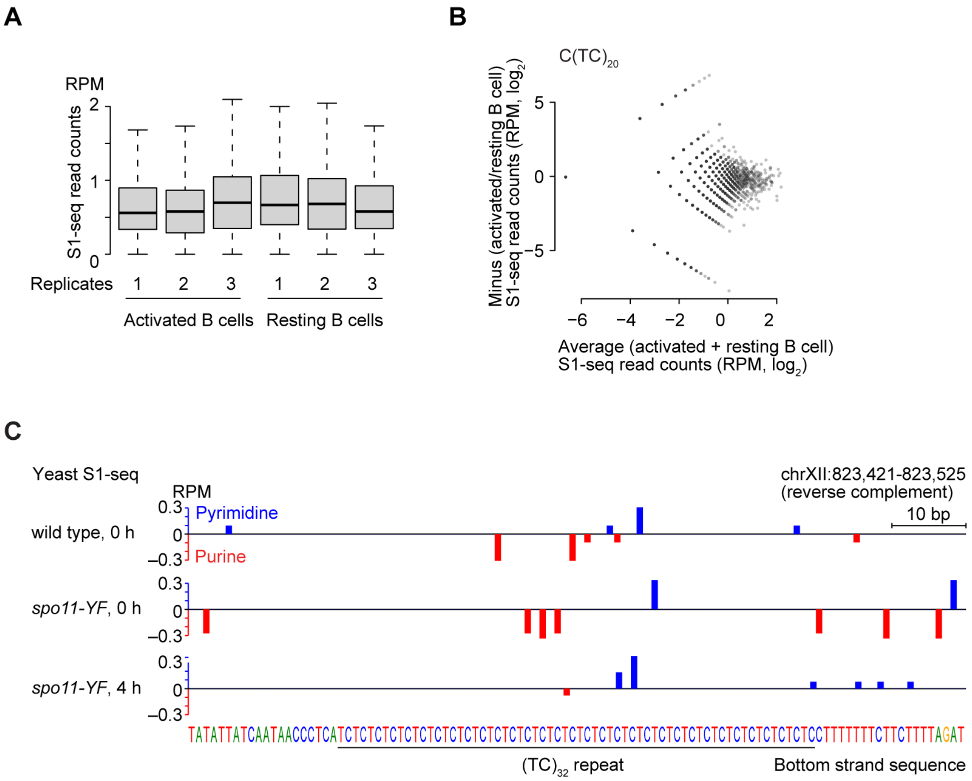

Fig. S6. S1-seq maps at H-DNA motifs in B cells and yeast.

(**A**) Similar S1-seq read counts at C(TC)$_{20}$ loci in activated vs. resting B cells. Box plots are as defined in the legend to Fig. S2*B*. Three biological replicates are shown for each cell state.

(**B**) MA plot of S1-seq read counts at C(TC)$_{20}$ loci comparing activated and resting B cells.

(**C**) S1-seq reads around the only yeast TC repeat longer than the minimum necessary for DISC signal in mouse (i.e., longer than C(TC)$_{17}$).

Table S1. S1-seq mapping statistics

Genotype and age	Read length	No. of reads	No. of reads mapped uniquely
Spo11$^{-/-}$, 12 dpp	PE100	22,056,481	14,496,927
resting B cells, adult	PE100	14,856,988	7,500,995
resting B cells, adult	PE100	16,678,604	8,801,390
resting B cells, adult	PE100	15,496,834	8,633,588
activated B cells, adult	PE100	15,309,467	8,911,754
activated B cells, adult	PE100	12,894,676	6,919,805
activated B cells, adult	PE100	15,149,694	8,586,425

Table S2. Genome assembly versions used in this study

Genbank common name	Scientific name	Genome version
house mouse	*Mus musculus*	mm10
Norway rat	*Rattus norvegicus*	rn6
Chinese hamster	*Cricetulus griseus*	criGriChoV2
Ord's kangaroo rat	*Dipodomys ordii*	dipOrd1
naked mole-rat	*Heterocephalus glaber*	hetGla2
thirteen-lined ground squirrel	*Ictidomys tridecemlineatus*	speTri2
American pika	*Ochotona princeps*	ochPri2, ochPri3
Sunda flying lemur	*Galeopterus variegatus*	galVar1
northern tree shrew	*Tupaia belangeri*	tupBel1
human	*Homo sapiens*	hg38
baker's yeast	*Saccharomyces cerevisiae*	sacCer2
gray mouse lemur	*Microcebus murinus*	micMur1, micMur2
Philippine tarsier	*Carlito syrichta*	tarSyr2
